# The characteristics of high-risk HPV-negative cervical cancer: a systematic review and meta-analysis

**DOI:** 10.3389/fonc.2024.1452834

**Published:** 2024-10-09

**Authors:** Jicheng Lou, Feng Guo

**Affiliations:** ^1^ Department of Gynecology and Obstetrics, The Central Hospital of Wuhan, Tongji Medical College, Huazhong University of Science and Technology, Wuhan, China; ^2^ Department of Rehabilitation Medicine, Tongji Hospital, Tongji Medical College, Huazhong University of Science and Technology, Wuhan, China

**Keywords:** high-risk human papillomavirus-negative cervical cancer, hr-HPV-negative cervical cancer, characteristics, systematic review and meta-analysis, cervical cancer

## Abstract

**Introduction:**

In order to comprehensive understanding of the characteristics of high-risk HPV-negative cervical cancer and aiming to provide certain evidence in the diagnosis and treatment of this kind of disease, the clinical and pathological characteristics of high-risk HPV-negative (hr-HPV-negative) cervical cancer was evaluated through systematic review and meta-analysis.

**Methods:**

We employed a systematic search strategy incorporating both MeSH terms and free words across multiple databases including PubMed, Embase, Cochrane Library, Web of Science, CNKI and Wanfang. The strategy was specifically designed to identify articles investigating risk factors or clinical characteristics of hr-HPV-negative cervical cancer. Our database search spanned from their inception until October 2023. The literature selection and data collection processes were guided by predetermined inclusion and exclusion criteria. Each included research was independently evaluated by two reviewers using the Newcastle-Ottawa Scale (NOS) and a meta-analysis was conducted utilizing RevMan5.4.

**Results:**

In total, nine studies published between 2013 and 2020 were included. The risk of lymph node metastasis was notably higher in hr-HPV-negative cervical cancer patients compared to hr-HPV-positive counterparts [RR=1.99, 95%CI (1.46–2.72), P<0.0001]. It was found that clinical staging of Ib2 or higher grade was significantly increased in hr-HPV-negative cervical cancer patients, as reported by seven studies [RR=1.42, 95%CI (1.15–1.77), P=0.0001]. Non-squamous carcinoma incidence, reported in seven studies, was significantly higher in hr-HPV-negative patients [RR=2.56, 95%CI (1.63–4.01), P=0.001]. Adenocarcinoma incidence was also significantly higher in hr-HPV-negative cervical cancer patients [RR=2.36, 95%CI (1.44–3.89), P=0.005].

**Conclusions:**

Cervical cancer patients not infected by hr-HPV exhibited increased rates of lymph node metastasis, adenocarcinoma, and non-squamous carcinoma. And they were often diagnosed at more advanced stages. These variables suggest that hr-HPV-negative cervical cancer patients presented with a more severe health status at the time of their initial cancer diagnosis.

## 1 Introduction

Cervical cancer, a commonly occurring gynecological malignant tumour, is typified by a high incidence and mortality rate (1). Extant data suggest that approximately 90% of cervical cancer cases can be ascribed to human papillomavirus (HPV) infection, predominantly high-risk HPV (hr-HPV) infection (2). Indeed, hr-HPV infection is a crucial factor in the initiation and progression of precancerous cervical lesions (3). Screening for hr-HPV has been proven to be an effective method in circumventing the invasive form of cervical cancer. Although hr-HPV is recognized as a necessary but insufficient prerequisite for cervical cancer, HPV screening has demonstrated superior capacity to detect persistent high-grade lesions at an early stage and provide a more substantial reduction in risk over an extended period, compared to cytology (4, 5). However, in younger women, HPV screening may lead to an overdiagnosis of cervical intraepithelial neoplasia (CIN II) (6).

Presently, studies on the relationship between hr-HPV infection and cervical cancer have gained significant attention. However, it is important to note that not all patients with cervical cancer are hr-HPV-positive. Clinical practice revealed the existence of hr-HPV-negative patients. This subgroup may face a delayed diagnosis due to their negative hr-HPV status, which can result in advanced-stage detection or poor prognosis (7, 8).

Cervical cancer continues to have a significant incidence, despite global efforts in HPV vaccination campaigns (9). Considering China’s vast population, even a relatively low percentage of hr-HPV-negative cervical cancer patients amounts to a significant number of individuals. Thus, to minimize the risk of missed diagnoses and to improve the prognosis and quality of life for these patients, it is imperative to give due consideration to hr-HPV-negative cervical lesions in clinical practice. So we need to fully understand the characteristics of the hr-HPV-negative cervical cancer.

Although numerous independent studies have identified risk factors associated with hr-HPV-negative cervical cancer, few meta-analyses have been conducted on these studies. Hence, this study employs a meta-analytical approach to systematically analyze the clinical and pathological characteristics of patients with hr-HPV-negative cervical cancer. The ultimate objective is to generate innovative insights that can inform clinical practice in cervical cancer treatment and enhance patient outcomes.

## 2 Methods

### 2.1 Search strategy

A computerized literature search was executed on databases including PubMed, Embase, Cochrane Library Web of Science, CNKI and Wanfang to identify studies concerning risk factors or clinical characteristics of hr-HPV-negative cervical cancer. The search strategy entailed an amalgamation of topic keywords and unrestricted terms. Every entry within these databases was retrieved, dating back to their inception until October 2023. The core search terms included: cervical cancer, cervical lesions, high-risk human papillomavirus, high-risk HPV, hr-HPV, negative, characteristics, risk factors.

### 2.2 Inclusion and exclusion criteria

#### 2.2.1 Inclusion criteria

Inclusion criteria included: (1) Studies designed such as case-control, retrospective, and cohort studies; (2) Patients diagnosed with cervical cancer by histopathological examination; (3) Subjects screened for HPV and definitively diagnosed as hr-HPV-negative; (4) Papers discussing the epidemiology, prevention, diagnosis and treatment of hr-HPV-negative cervical cancer; (5) Literature providing full text and complete data; (6) Patients not in the pregnancy period; (7) Patients without autoimmune disease; (8) Patients without a history of hysterectomy and cervical surgery.

#### 2.2.2 Exclusion criteria

Exclusion criteria comprised: (1) Duplicate publications; (2) Foundational literature or reviews; (3) Literature where full-text could not be obtained; (4) Literature with unclear outcome indicators; (5) Literature encompassing abstracts, case reports, and meeting minutes; (6) Literature providing incomplete or inaccurate clinical data.

### 2.3 Data extraction and quality assessment

Upon completion of the literature search, Endnote 20 was utilized to review and eliminate articles including repeated publications and irrelevant researches. Two independent reviewers meticulously scrutinized and cross-verified the selected literature based on the stipulated inclusion and exclusion criteria. Following a preliminary screening of titles and abstracts, the full content of potentially relevant papers were thoroughly read to finalize inclusion. In case of disagreements between the two reviewers, a third reviewer was consulted. Data extracted from the included literature comprised: (1) Title, author, year of publication, total sample size, hr-HPV-negative sample size, and hr-HPV-positive sample size; (2) Research characteristics such as the age of study subjects and relevant study factors. The quality of the selected publications was evaluated using the Newcastle Ottawa Scale (NOS) by the two independent reviewers. Criteria for evaluation included case selection, case representativeness, control selection, comparability of case and control groups, exposure analyses and follow-up integrity. Papers scoring 1 to 3 points were deemed poor quality, 4 to 6 points medium quality, and 7 to 9 points high quality. In case of subjective selection bias, two reviewers required to discuss, or a third reviewer was to be consulted.

### 2.4 Statistical analysis

The meta-analysis was performed using RevMan5.4 software. For count data, the risk ratio (RR) was used as the effect index. The corresponding 95% confidence interval (CI) was then calculated and expressed via a forest plot. The degree of heterogeneity among the articles was ascertained using I^2^ and Q statistics. The dichotomous outcomes were analyzed using the fixed-effects model. Random-effects model was used when the heterogeneity was greater than 50%. I² > 50.0% or P < 0.10 was considered to be significant heterogeneity. Then sensitivity analyses were conducted to assess the robustness of the pooled conclusions by using the fixed effects model. In our research, we calculated the fixed-effects model and random-effects model at the meantime in all the forest map. P < 0.05 indicated statistical significance.

## 3 Results

### 3.1 literature search results

**Figure 1:**
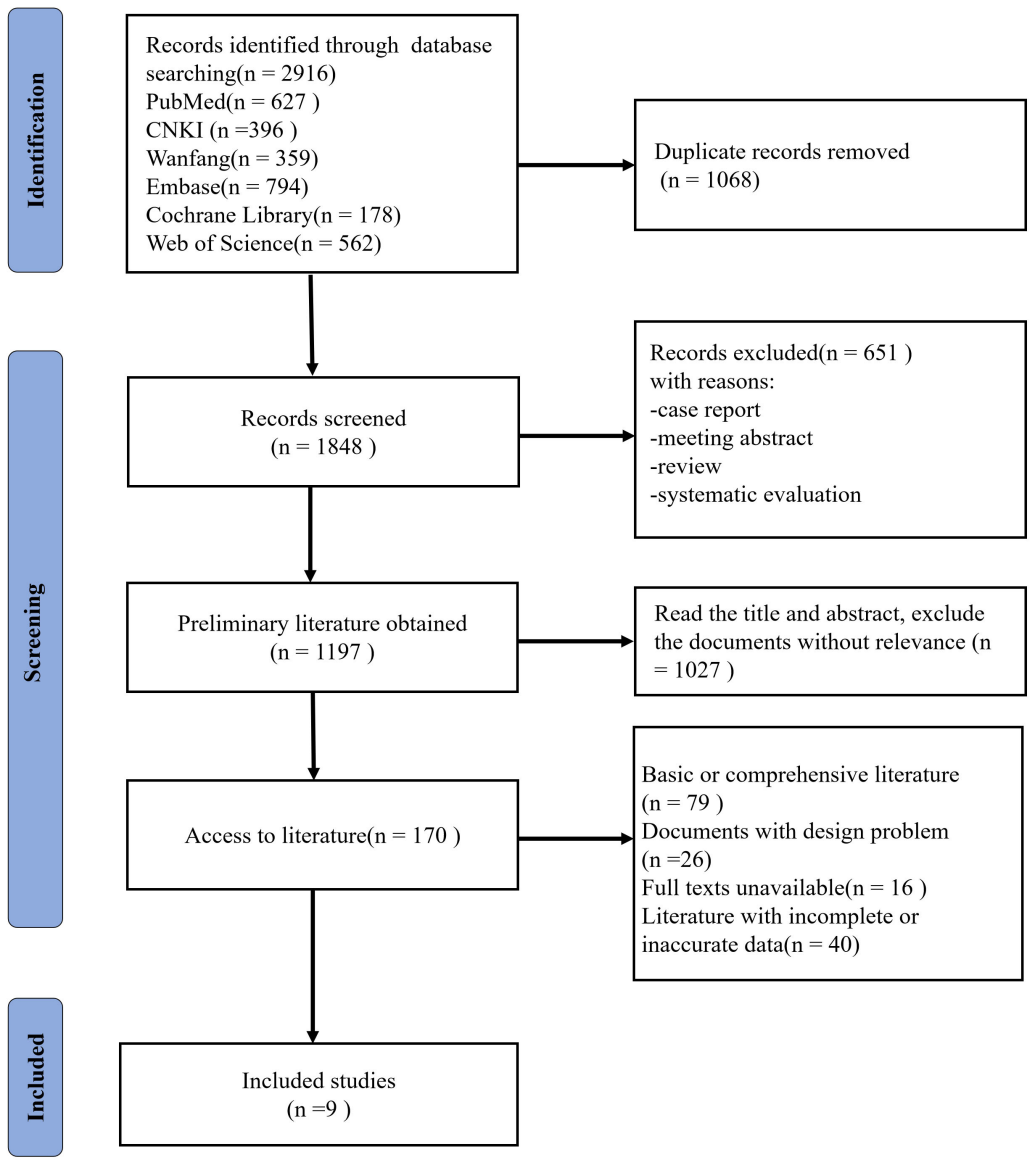
Flow diagram of article selection according to the PRISMA guidelines.

Initially, 2916 records were retrieved, which reduced to 1848 following the removal of 1068 duplications. 651 records were excluded with reasons of case report, meeting abstract, systematic evaluations or review. Upon perusing the titles and abstracts, 1027 articles that lacked apparent relevance were discarded, resulting in a total of 170 articles. Further scrutiny led to the exclusion of 79 foundational class articles or reviews, 26 papers with unresolvable design issues, 16 records with full texts unavailable, 40 documents with incomplete or inaccurate clinical data. Ultimately, nine articles were deemed suitable for inclusion (10–18). **Figure 1** illustrates the steps involved in the literature review.

### 3.2 Basic characteristics and quality assessment of included literature

The analysis encompassed nine articles published between 2013 and 2020, with a collective total of 3916 patients, 672 hr-HPV-negative and 3244 hr-HPV-positive. The Newcastle Ottawa Scale (NOS) was employed to gauge the quality of the included articles. **Table 1** provides a summary of the included studies and an evaluation of their respective quality.

**Table 1:** Main characteristics and NOS scores of the studies included in the meta-analysis.

Author	Year	Country	Study design	Sample size	hr-HPV negative	hr-HPV positive	NOS
Baalbergen (10)	2013	Netherlands	Retrospective	113	12	101	5
Rodríguez (11)	2015	Spain	Retrospective	136	8	128	6
Omori (12)	2015	Japan	Retrospective	44	14	30	8
Okuma (13)	2016	Japan	Retrospective	71	13	58	5
Banister (14)	2017	Canada	Retrospective	261	20	241	7
Lei (15)	2018	Sweden	Retrospective	2845	552	2293	8
Nicolás (16)	2019	Spain	Retrospective	214	21	193	6
Chong (17)	2019	Korea	Retrospective	30	18	12	4
Kaliff (18)	2020	Sweden	Retrospective	202	14	188	5

### 3.3 Results of meta-analysis

#### 3.3.1 Meta-analysis of positive lymph node metastasis

**Figure 2:**
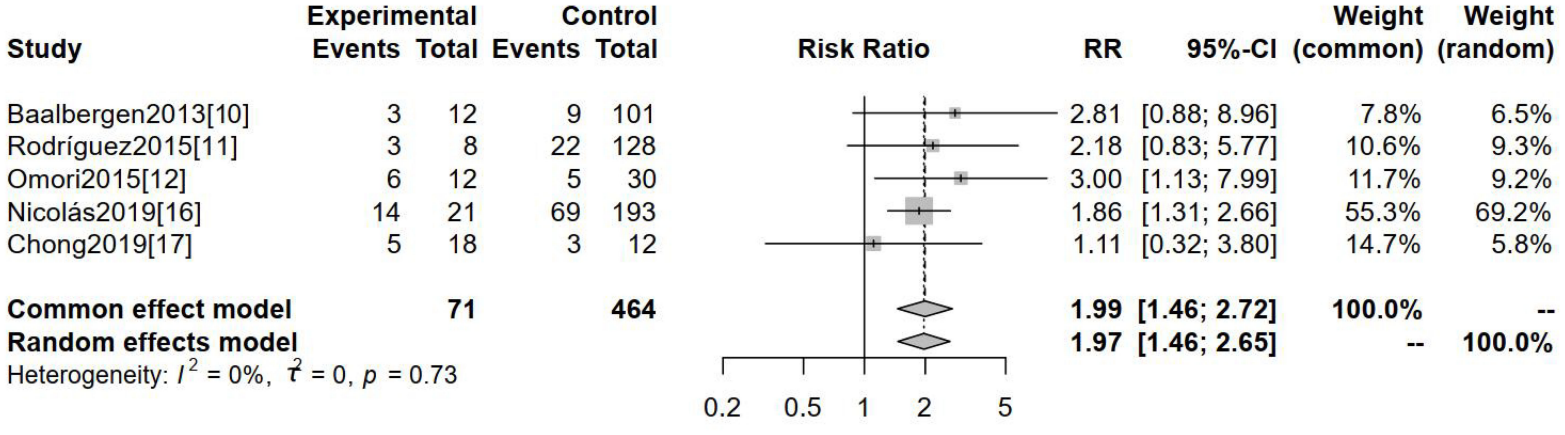
Comparison of positive lymph node metastases between hr-HPV-negative and -positive cervical cancer.

Five separate studies assessed positive lymph node metastasis as a potential outcome indicator. **Figure 2** indicates a substantial divergence in the positive rate of lymph node metastasis between hr-HPV-negative and hr-HPV-positive cervical cancer patients [RR=1.99, 95%CI (1.46-2.72), P<0.0001]. This statistically significant discrepancy serves as evidence.

#### 3.3.2 Meta-analysis of clinical staging

**Figure 3:**
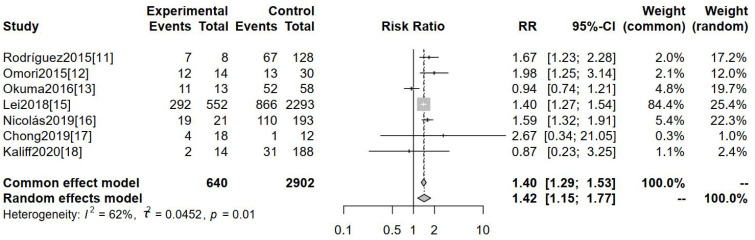
Comparison of the clinical staging ≥Ib2 of cervical cancer in patients with hr- HPV-negative and -positive.

Seven studies deployed clinical staging ≥Ib2 as an outcome measure. Clinical stages in studies included were all based on FIGO 2009. **Figure 3**, featuring the forest map analysis, reveals a significant difference in clinical staging ≥Ib2 between hr-HPV-negative and hr-HPV-positive cervical cancer patients [RR=1.42, 95%CI (1.15-1.77), P=0.0001].

#### 3.3.3 Meta-analysis of incidence of non-squamous carcinoma

**Figure 4:**
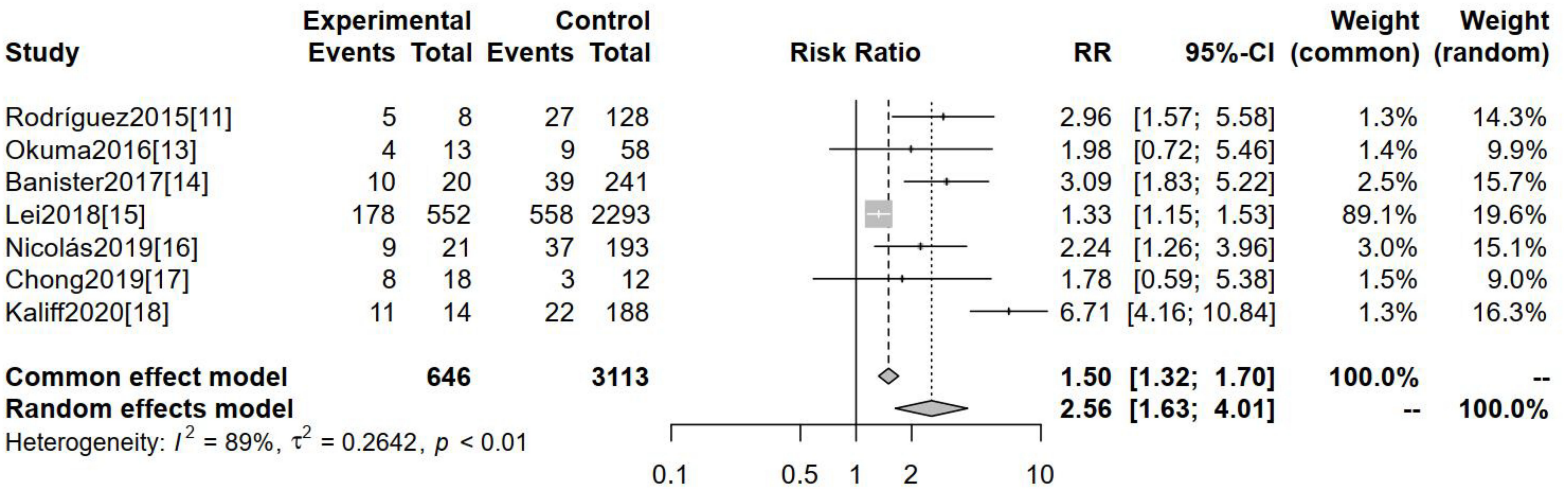
Comparison of the incidence of non-squamous carcinoma in hr-HPV-negative and -positive cervical cancers.

The incidence of non-squamous carcinoma was evaluated as an outcome indicator across seven studies. **Figure 4** demonstrates a statistically significant difference in the incidence of non-squamous carcinoma between these two groups [RR=2.56, 95%CI (1.63-4.01), P=0.001].

#### 3.3.4 Meta-analysis of incidence of adenocarcinoma

**Figure 5:**
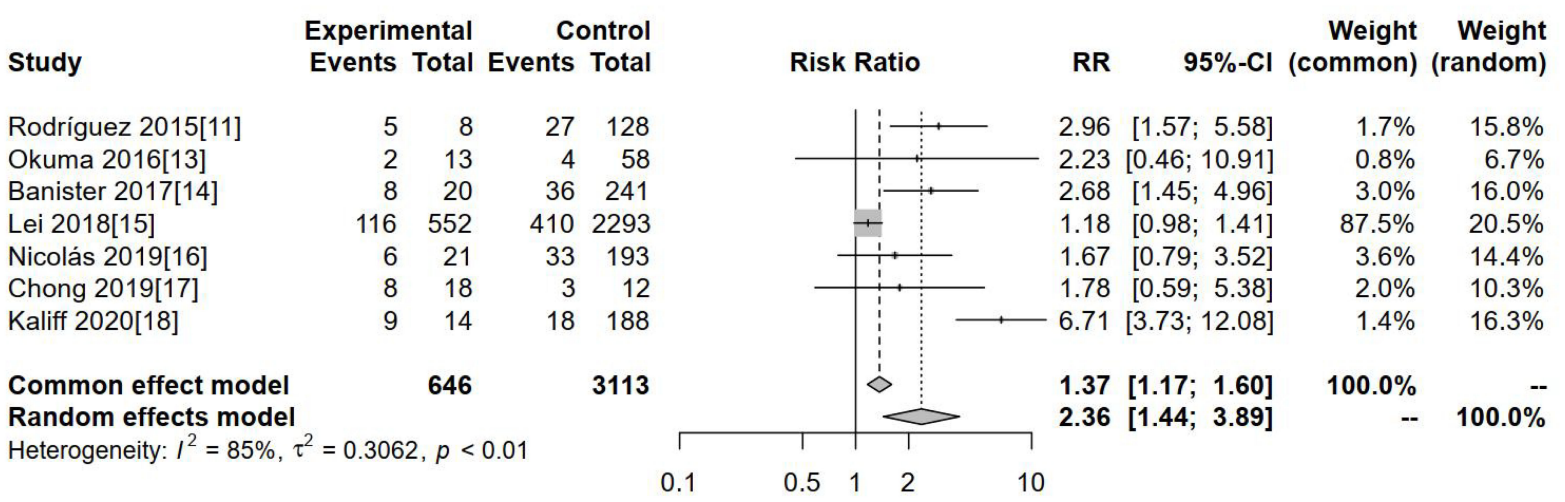
Comparison of the incidence of adenocarcinoma in hr-HPV-negative and -positive cervical cancer cases.

Seven studies investigated the incidence of adenocarcinoma as an outcome indicator. The forest map results, presented in **Figure 5**, indicate a significantly higher incidence of adenocarcinoma in hr-HPV-negative cervical cancer patients compared to those testing hr-HPV-positive [RR=2.36, 95%CI (1.44-3.89), P=0.005].

### 3.4 Publication bias analysis

**Figure 6:**
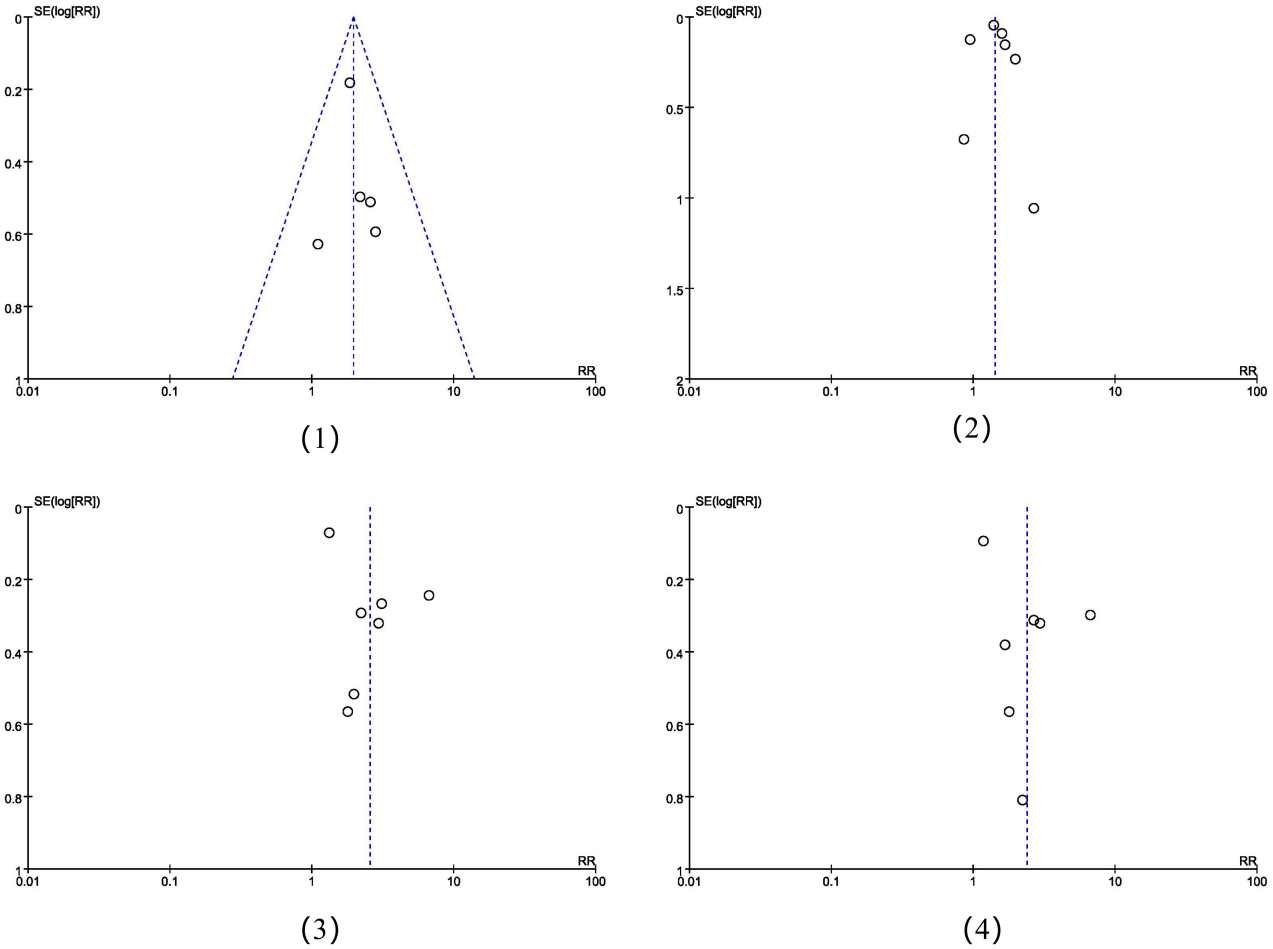
The funnel plots of the four meta-analysis placed in sequential order: (1) positive lymph node metastasis, (2) clinical staging ≥Ib2, (3) the incidence of non-squamous carcinoma, (4) the incidence of adenocarcinoma.

The funnel plots showed that there was no obvious risk of literature publication bias in the meta-analysis of positive lymph node metastasis and clinical staging ≥Ib2, while the risk was exisit in the incidence of non-squamous carcinoma and adenocarcinoma(**Figure 6**). The risk of bias could have arisen from various factors including the limited sample size of the studies, subpar quality of the sourced papers, among other influences.

## 4 Discussion

The global incidence of high-risk HPV infections and cervical cancer poses a significant threat to the health and quality of life of women, engendering profound psychological stress and economic burdens. HPV vaccines have shown potential to decrease the prevalence of hr-HPV infections and associated cervical lesions (19). Nonetheless, not all instances of cervical cancer are initiated and progressed through HPV infection, approximately 5.5-11.0% of cervical cancer pathology has been proven to be HPV-negative upon detection (20, 21).

Evidence posits a robust correlation between hr-HPV infection and cervical squamous carcinoma, whereas a linkage between hr-HPV infection and cervical adenocarcinoma has been discerned but has yet to achieve statistical significance (22). Certain studies have suggested that the formalin fixation of tissues can instigate alterations in the high-frequency sequences of genes, potentially resulting in false negatives, thereby reducing the detection rate of PCR (23). However, researches also found that some rare pathological types of cervical cancer with poor prognosis were truly HPV-negative (24, 25).

As such, it is of paramount importance to administer thorough care to individuals presenting with clinically hr-HPV-negative cervical lesions. In consideration of the above, the current study employs a meta-analytical approach to explore the clinical features, pathological characteristics, and associated factors in patients with hr-HPV-negative cervical cancer. The aim is to generate innovative concepts for clinical guidance in the treatment of cervical cancer, ultimately enhancing the prognosis for individuals diagnosed with the condition.

The study encompassed a total of nine publications, predominantly spanning from 2013 to 2020. The findings demonstrated that patients with hr-HPV-negative cervical cancer manifested a risk of lymph node metastasis 1.99 times greater than that in patients with hr-HPV-positive cervical cancer [RR=1.99, 95%CI (1.46-2.72), P<0.0001]. This could be attributed to the fact that certain cervical cancers, not instigated by the HPV virus, do not demonstrate typical progression or manifestation (26). By the time such illnesses are detected, they have likely advanced, thus posing a significant risk of lymph node metastasis.

In relation to clinical staging, individuals with hr-HPV-negative cervical cancer were 1.42 times more likely to be staged at greater than Ib2 compared to their hr-HPV-positive counterparts [RR=1.42, 95%CI (1.15-1.77), P=0.001]. The early detection of hr-HPV-negative cervical cancer is challenging, often leading to late-stage diagnosis, possibly bypassing the optimal window for surgical intervention (27). This late discovery often results in a poor prognosis and reduced survival rates.

With respect to non-squamous cancer, patients with hr-HPV-negative cervical cancer were found to have a 2.56 times greater incidence rate compared to patients with hr-HPV-positive cervical cancer [RR=2.56, 95%CI (1.63–4.01), P=0.001]. However, gastric adenocarcinoma and other pathological types remain relatively rare among hr-HPV-negative cervical adenocarcinomas. Current preventative measures and treatment options for hr-HPV are predominantly effective against squamous carcinoma and squamous epithelial lesions, which are primarily driven by HPV. Cervical gastric adenocarcinoma represents a separate entity with a clinically aggressive demeanor (28, 29). The risk of adenocarcinoma was 2.36 times higher in patients with hr-HPV-negative cervical cancer compared to those with hr-HPV-positive cervical adenocarcinoma [RR=2.36, 95%CI (1.44–3.89), P=0.005]. This could be attributed to the fact that hr-HPV infection may not contribute to the etiology of adenocarcinoma and other uncommon pathological types of cervical cancer (30).

A review found that the HPV positive rate for the period 1990-1999, 2000-2005 and 2006-2010 was 85.9%, 87.9% and 92.9%, respectively (31). The decrease in HPV negative cancers is likely to reflect improvements in the HPV detection methods (32). It also shows that the hr-HPV negative cervical cancers may contain false negative cases. Sampling errors including inadequate cellularity, obscuring blood, inflammation and cytolysis may lead to false negative. In addition, samples stored for more than 30 years have a significantly lower HPV detection rate (24). Banister et al. found that there is a subset of tumors, which no longer express HPV E6/E7 oncogenes (HPV-inactive). These HPV-inactive tumors have a global decrease in DNA methylation and an increased WNT/β-catenin and Sonic Hedgehog signaling, which may decrease the HPV positive detection rate (14). And a negative test result may occur if the test does not detect a specific rare hr-HPV type. More advanced detection methods will improve the accuracy of the detection, for instance, a study initiated by Cancer Genome Atlas Research Network used next-generation sequencing to characterize primary cervical cancers, found that only 5% of cervical cancers were HPV-negative (33). HPV vaccination and HPV screening can not prevent or detect the hr-HPV negative cervical cancers (32). Therefore, how to detect and prevent hr-HPV negative cervical cancer will become an important study.

Despite yielding some clinically relevant findings that could contribute to evidence-based therapeutic counseling and treatment, this study also has its limitations: The limited number of articles included may impact the comprehensiveness of the meta-analysis. Potential biases might have arisen since all the included papers were retrospective studies. Due to the detection technique, there might be false negative hr-HPV diagnoses, adding the risk of bias. In the future, we will strive to incorporate high quality and multi-center research results into our work.

## 5 Conclusion

In conclusion, patients with hr-HPV-negative cervical cancer are typically diagnosed at later stages compared to those with hr-HPV-positive cancer. Furthermore, the incidence of adenocarcinoma, non-squamous malignancies, and lymph node metastases were found to be higher among hr-HPV-negative cervical cancer patients, indicating a more advanced disease stage at diagnosis.

## Data Availability

The original contributions presented in the study are included in the article/supplementary material. Further inquiries can be directed to the corresponding author.
